# An Observational Study on the Walking Proximity between Off licenses plus Bookmakers and Community Mental Health Facilities in County Dublin

**DOI:** 10.1192/j.eurpsy.2022.871

**Published:** 2022-09-01

**Authors:** M.C. Wong, Z. Azvee, C.W. Wong, R. Duffy

**Affiliations:** 1 Connolly Hospital Blanchardstown, Department Of Psychiatry, Dublin, Ireland; 2 Mater Misericordiae University Hospital, Department Of Psychiatry, Dublin, Ireland; 3 The Chinese University of Hong Kong, Department Of Computer Science And Engineering, Shatin, Hong Kong PRC

**Keywords:** GIS, gambling, community mental health service, alcohol

## Abstract

**Introduction:**

Dual diagnosis is commonly treated by Community Mental Health Team (CMHT). Addiction is a common complicating factor in individuals with major mental illnesses. It is established that businesses on high streets impact on the public’s health.

**Objectives:**

We hope to generate discussion about the planning and the placement of community mental health services.

**Methods:**

The location of County Dublin community mental health teams’ outpatient clinics’ and day hospitals’ were obtained from the Health Service Executive directory website. All off licenses’ and bookmakers’ addresses in County Dublin were obtained from the Irish Revenue Commissioners website. The distances were measured using Google Maps and a programming script to generate a matrix under one-kilometre radius walking distances between the locations. No ethical approval is required. All Data are sought from publicly available websites.

**Results:**

On average, there are 6.29 (SD 4.20; Median 5.) off-licenses and 2.4 (SD 2.28; Median 2) bookmarkers offices per mental health facility within1 km walking distance. The Central Dublin Mental Health Service has the highest prevalence of off-licenses (45, 34.4%), and the Central South Dublin Service(20, 39.2%) has the highest prevalence of bookmakers. Southeast Dublin Service has the lowest in both businesses. The closest distance to an off-license from mental health facilities was 0 meters.

**Conclusions:**

Psychiatrists have a role in advocating the needs of individuals with dual diagnoses. The Department of Health and Health Service Executive (HSE) should develop a guideline and protocol for the community health services in the structuring and planning mental health services in the community health outpatient service setup.

**Disclosure:**

No significant relationships.

